# Can Rehabilitative Travel Mobility improve the Quality of Life of Seasonal Affective Disorder Tourists?

**DOI:** 10.3389/fpsyg.2022.976590

**Published:** 2022-09-28

**Authors:** Sha Sha, Wencan Shen, Zhenzhi Yang, Liangquan Dong, Tingting Li

**Affiliations:** ^1^Health and Rehabilitation School, Chengdu University of Traditional Chinese Medicine, Chengdu, Sichuan, China; ^2^Tourism School, Sichuan University, Chengdu, China; ^3^Shenzhen Tourism College, Tourism College of Jinan University, Jinan University, Guangzhou, Guangdong, China; ^4^College of Economics and Management, Xinjiang Agricultural University, Ürümqi, Xinyang, China

**Keywords:** rehabilitative travel mobility (RTM), tourist health, quality of life (QOL), seasonal affective syndrome (SAD), physical health, mental health, social health

## Abstract

Rehabilitation mobility has become a new demand and travel mode for people to pursue active health. A large number of tourists choose to escape the cold in warm places to improve their health every winter. In this study, we collected the health index data of Seasonal Affective Disorder (SAD) tourists from western China before and after their cold escape in Hainan Island in winter, aiming to compare whether rehabilitating cold escape can improve the Quality of Life (QOL) of SAD tourists by hierarchical analysis. Compared with previous studies, this paper has the following contributions: Firstly, the study samples were accurately screened according to the pathogenesis of SAD tourists and the confounding factors were strictly controlled; Secondly, the observational experimental method was used to conduct inter-group and intra-group control studies on 695 samples, and the results were more objective and reliable. Thirdly, the effect of treatment on the quality of life (QOL) of 397 tourists in the rehabilitation mobility group was quantitatively evaluated from three factors including age, gender and sunshine exposure level by multivariate analysis of variance. Research results show that the rehabilitation environment brought by rehabilitation activities can help improve the health status of tourists. Therefore, this paper proposes the concept of “Tourism Therapy” and constructs a theoretical framework. The conclusion of this paper provides a scientific basis and reference for the study of tourism healing as a non-medical alternative therapy.

## Introduction

As the pace of life accelerates, people generally have some psychological problems such as depression and anxiety (Wang, 2018). Seasonal Affective Disorder (SAD), also known as “seasonal depression,” is a kind of continuous strong sadness or emotional disorder caused by seasonal changes (American Psychiatric Association, 2013). This feeling will strongly affect tourists’ behavior and attitude (Nimrod et al., 2012), and even prevent tourists from engaging in leisure activities (Dupuis and Smale, 1995). With the increase of work and life pressure, sub-health groups with SAD symptoms have gradually become a common phenomenon (Blazer, 2005). SAD occurs in two seasons, one from late autumn to early winter, which is called “winter depression,” and the other from late spring to summer, which is called “summer depression” (Pjrek et al., 2016). It happens more commonly in winter than in summer (Yildiz et al., 2016). People with SAD are in a good state of health for most of the year, but they feel depressed in winter or summer (American Psychiatric Association, 2013). The study found that the incidence of SAD is significantly related to the average temperature and the length of a light cycle in the same month, and the light exposure has a significant influence on seasonal emotional fluctuations (Rosenthal et al., 1986). Nimrod et al. (2012) pointed out that people with SAD symptoms were more eager to sunbathe, and the less outdoor leisure activities they participated in, the more depressed they were.

The occurrence rate of SAD is significantly related to the average temperature of the month and the length of the light cycle, and depression occurs regularly in winter (Rosenthal et al., 1986). SAD is also common in mid-latitude areas with mild winters, such as Seattle and Vancouver. People living within the Arctic Circle are particularly susceptible to polar nights because of them (Rosen et al., 1990). In addition, prolonged cloudy days can also aggravate the symptoms of SAD (Jankowski, 2017), which is 4 times higher in women than in men. Approximately 11 million people worldwide have been identified with SAD (Magnusson, 2000). The prevalence of SAD in different age groups is not yet clear, but it appears to be higher in adults than in the elderly or in children and adolescents (Lam et al., 2001). SAD usually starts at the age of 20–30 years (Lam et al., 2006). Some studies have found that the prevalence of SAD is higher in higher latitudes, but the detection of cases depends on screening tools (Levitan, 2007). Theories about the cause of SAD are still controversial. Some scholars believe that sunlight has a great relationship with seasonal emotional fluctuations of the human body, rather than temperature (Sarran et al., 2017). Modern medical mechanisms use SAD as the simultaneous injury of biological rhythm and mood of the body (Wehr et al., 2001). Some scholars believe that SAD is related to the lack of serotonin, pointing out that full-spectrum artificial light can improve the situation by stimulating the production of serotonin (Liu et al., 2019). Some scholars also believe that melatonin produced by the pineal gland is the main cause of SAD, because there is a connection between pineal gland and retina. Melatonin secretion is greatly affected by light and is an important zeitgeist in the endocrine system (Danilenko et al., 1994). Subsequent studies have confirmed that the pathological mechanism of SAD includes changes in physical rhythm and photoperiod (Lewy et al., 2006).

At present, there are three commonly used treatments: Light Therapy, Drug Therapy, and Psychotherapy (Rosenthal et al., 1983). Light Therapy is widely recognized for its minimal side effects (Dubovsky, 2015). However, the application scenarios of “light therapy” in existing studies are only limited to hospitals and indoors, and few studies pay attention to the therapeutic mechanism of sub-health tourists in RTM. The research on tourism rehabilitation mechanism based on RTM can help to provide corresponding theoretical knowledge for disease prevention of healthy people and health recovery of sub-health people. Therefore, based on the perspective of mobility, this paper focuses on the relationship and impact between RTM and tourist health. The population of this study are SAD winter symptom tourists. This paper quantitatively evaluates the therapeutic effect of RTM on the Quality of Life (QOL) of this group, and further verifies the tourism therapeutic function in order to take tourism as a non-medical intervention means to improve the QOL of tourists.

## Literature review

### Theory of rehabilitation mobility tourism

The development of global integration has promoted the study of mobility. Mobility interweaves people, places, things, and relationships into the way the world works. Schiller and Salazar (2013). People’s use of service facilities, social interaction with people around them, perception and interaction with the environment all inevitably change on each specific spatial and temporal scale (Gatrell and Anthony, 2013). Mobility, from the perspective of social science, tends to study the social phenomenon of human activities, focusing on the complex interaction between subject and object in the Mobility context (Sheller and Urry, 2006). The COVID-19 pandemic has disrupted the Mobility of people, but released a new trend (Xiong et al., 2022). A new trend is Rehabilitation Tourism Mobility (RTM), which is to release physical and mental pressure and actively pursue active health. Gatrell and Anthony (2013) proposed the concept of “Rehabilitative Tourism Mobility (RTM).” He believes that connections between people and places can be gained through mobility. Since then, researchers have begun to pay attention to the positive effects of RTM on human physical and mental health (Gatrell and Anthony, 2013; Coghlan, 2015). Xu and Wang (2018) drew on the concept of “rehabilitation flow” in health geography to explain the internal mechanism of RTM in promoting health. The theory of RTM provides a theoretical basis for explaining tourism mobility, seasonal flow, and long-term migration flow in the life process. Kou et al. (2017) revealed the health experience of seasonal retired floating population in Sanya, Hainan. The results show that the seasonal movement of destinations can only maintain the temporary and superficial physical and mental health of retirees, and it is difficult for them to rebuild their lives and achieve self-continuity. With the increase of various health problems and the continuous improvement of their own active health literacy, the role of RTM in the treatment of physical and mental health of tourists has become increasingly prominent.

Although RTM theory studies explain the relationship between mobility and individual health, there are still some limitations. Firstly, there is still a lack of relevant mechanisms to explain the effect of RTM on the physical and mental health of tourists. In the interaction with the non-habitual environment of RTM, tourists may get positive psychological hints and physical and mental pleasure (Zhang, 2009). At the same time, they may also be affected by some negative factors, such as foreign identity and regional cultural differences, resulting in unpleasant feelings such as fear and danger (Gatrell, 2011). Up to now, the mechanism of how RTM produces this kind of rehabilitative effect on tourists’ body and mind has not been revealed. Secondly, the existing researches on RTM do not highlight the particularity of the population, only focus on healthy people and ignore the unique characteristics of sub-health tourists, and the spillover of relevant knowledge is less. Therefore, this study on the impact of RTM on the physical and mental healing of sub-health tourists will help to promote the establishment of relevant theories.

### Quality of life theory

Medicine and psychology consider quality of life as a health-related and multidimensional concept, referring to patients’ subjective perceptions of the physical, psychological, and social effects of disease and treatment on daily life (Huang et al., 2020). Scholars have clearly embodied the improvement of human Quality of Life (QOL) into three dimensions: physical health, mental health, and social health (WHO, 1978). This concept is the same as the definition of health, which includes good physical health, mental state, and social adaptability of individuals (Chonghua, 1999). The World Health Organization (WHO) points out that health is not only the absence of diseases in the body but also mental health and good adaptability to society(Costanza et al., 2007), that is, people are in a good state in the three aspects of physiology, psychology and society, which just constitute the three dimensions of human QOL. Therefore, Quality of Life (QOL) has become an important indicator widely used to evaluate human health (Yulan, 2015).

In conclusion, the global spread of COVID-19 has raised concerns about mobility and human health. Relevant studies should pay more attention to the rehabilitation mobility to meet the health needs of tourists, to help people achieve safe and healthy travel (Xiong et al., 2022). Although many researchers have tried to touch on the prevention of disease infection and epidemic control in the process of travel (Zhenzhi et al., 2022). However, there is still a lack of interdisciplinary theoretical knowledge spillover on the mechanism of RTM to improve the Quality of Life (QOL) of human beings.

## Research design

### Research framework

**Figure 1:**
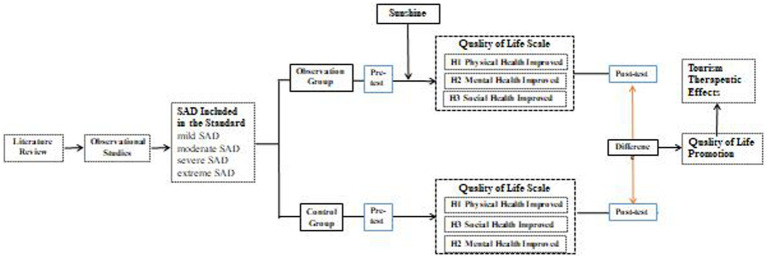
The basic framework of the research.

This paper focuses on the sub-health tourist group with SAD and seasonal RTM needs. They are characterized with distinct seasonal travel features. In winter, they travel from northern China to the humid and sunny southern China. In summer, they travel from southern China to the north to escape the cold. For the sake of health, SAD tourists usually choose a suitable residence during RTM to avoid the cold and summer heat to obtain physical and mental health (Dong and Wang, 2006). In order to clearly quantify the therapeutic effect of travel mobility on SAD tourists to escape the cold, the research framework of this paper is shown in Figure 1. The first step is to establish the inclusion and exclusion criteria of SAD tourist sampling based on the relevant literature and the previous research of scholars, and then conduct the hierarchical inclusion of SAD symptoms; The second step is to carry out a formal observational experiment to test the reliability and validity of the scale by randomly testing the health evaluation of SAD tourists who travel to escape the cold; The third step is to compare and analyze the differences of 20 indicators in the three dimensions of physical health, mental health and social health between the observation group and the control group and between the pre-test and post-test.

### Hypothesis

Does RTM have a therapeutic effect? Can the supportive surroundings of rehabilitative travel therapy improve QOL of tourists? Based on the above research, this paper selects SAD tourists who travel to Hainan Island in winter as the research object, puts forward the following three hypotheses, and discusses and verifies the impact of tourism rehabilitation on tourists in RTM.

*H1*: RTM has a therapeutic effect on the physical health of SAD tourists;

For a long time, foreign research on tourism activities and human physical health has focused on the health effects of individual tourists in system, organ, muscle energy, five senses, five internal organs, sleep and so on. The natural world is thought to be perceived through the experience of vision, hearing, taste, touch, and smell (Gong, 2018). In 1962, German scientist K. Franke found that being in the natural environment of travel would help the human body consciously adjust the balance nerve and restore the body rhythm (Zhao and Liu, 2006). Ohe et al. (2017) evaluated the body relaxation effect of forest travel therapy on the human body with multidisciplinary methods. Shan and Yao (2015) demonstrated that the health preservation of hot spring spa had a therapeutic effect on the body function of tourists. Excellent rehabilitative landscape is conducive to reducing the probability of tourists suffering from respiratory and cardiovascular diseases and promoting human physical health (Praschak-Rieder and Willeit, 2003). Therefore, this paper attempts to verify H1.

*H2*: RTM has a therapeutic effect on the mental health of SAD tourists;

From the perspective of positive psychology, scholars have verified that tourism can enhance tourists’ well-being. Ioannidou et al. believed that travel had a debilitating effect on mild depression in which tourists feel isolated, sad, fearful, and useless for a long time (Ioannidou, 2005; Liu and Cui, 2013) verified the impact of tourism on the improvement of human anxiety and depression with Hamilton Anxiety Scale (HAMA). In addition, studies have found that both seaside and forest tourism can make tourists more stable, reduce the sense of stress in the groups with high depression level and have a positive impact on health (Bowler et al., 2010). Therefore, this paper attempts to verify H2.

*H3*: RTM has a therapeutic effect on the social health of SAD tourists;

Tourism encourages tourists to discover another true self, enhance understanding and contact with others, improve social communication ability and team consciousness and more cherish feelings (Wheeler et al., 2012). Voigt et al. (2013) argued that tourism provided an opportunity for tourists to learn about the world and society, and understand the relationship between people and society. Tourists also had chances to participate in social activities and establish new social relations, which theoretically helped to promote the realization of tourists’ individual social health (Lee and Yen, 2015). Therefore, this paper attempts to verify H3.

### Establish inclusion and exclusion criteria

According to relevant literature records, as winter comes, SAD patients will feel sleepy and anxious in the daytime (Manfredo et al., 1996), fatigue or declined in energy and their sleep will increase (Blashko et al., 1999). There will be some difficulties in concentrating attention. Appetite will increase (especially sweets and carbohydrates) leading to weight gain (Roecklein et al., 2013). Therefore, the inclusion index of the samples is set as: tourists whose body fluctuate and change in six aspects of sleep, weight, appetite, energy, emotion and social interaction due to the reduction of sunshine in their usual place of residence in winter. The numbers of 0, 1, 2, 3, and 4, respectively, represent five grades of the SAD winter symptoms, namely not, mild, moderate, severe and extreme. The lower the score, the lighter the disease. Scores of 10+ will be included in SAD winter symptoms. Every 5 points is put into one grade. The higher the score, the more serious the SAD winter symptoms. See Table 1 for the score indicators. The exclusion criteria: (1) tourists under 18 years old; (2) Non-SAD tourists with scores less than 10 points; (3) Visitors from non-sampling areas; (4) tourists with general underlying diseases or during medication.

**Table 1:** Scoring criteria table of winter SAD tourists inclusion.

Index	Specific items	Score	Inclusion criteria
		*A* = 5 *B* = 4 *C* = 3 *D* = 2 *E* = 1	*X* ≥ 10 is included
Physical state	Sleep quality change	A. Extremely bad (no interest)	0 level:0<*X* ≥ 10:not SAD
	Fatigue change	B. Not good (no interest)	1 level:10<*X* ≥ 15:mild SAD
	Appetite change	C. No change	2 level:15<*X* ≥ 20:moderate SAD
Mental state	Attention change	D. Good (Interested)	3 level:20<*X* ≥ 25:severe SAD
	Interests Changes	E. Very good (interested)	4 level:25<*X* ≥ 30:extreme SAD
Social state	Social situation change		

## Research method

### Sample-size calculation

The sample size of the observation group was similar to that of the control group, and the sample size was estimated by the completely randomized design method of comparison of the mean of the two samples in this study. The sample size was estimated according to the total score of the professional QOL test table.


$$N1=N2=\frac{2{\left(Z_{1-\alpha /2}+Z_{\beta }\right)}^{2}\sigma^{2}}{d^{2}}$$

The test level was defined as *α* = 0.05, *β* = 0.10, the power (1−*β*) was 90%, *Z*_1−*α*/2_ = 1.960, Z*_β_*=1.282, *σ* was the standard deviation or its estimate, *d* was the difference between the two means, and the sample size of the two groups was defined as *N*1 ≈ *N*2. According to the pre-experiment, the difference between the two populations was *D* = 83.45–76.98 = 6.47, and the larger standard deviation of *σ* was 7.03, which was substituted into the formula to calculate *N*1 = *N*2 ≈ 25 cases. In order to control the effect of loss to follow-up rate, the final sample size was estimated to be about 60 cases, that is, 30 cases in the observation group and 30 cases in the control group. Considering that this study is an observational experimental study with many interference factors and uncontrollable factors, in order to reduce research bias and strengthen the accuracy and credibility of the study, the sample size of the observation group and the control group is planned to be 10 times of *N*1 and *N*2, that is, no less than 300 cases in the observation group and no less than 300 cases in the control group.

### Case selection

Affected by geographical latitude and other factors, Chengdu and Chongqing are cold and humid in winter with few sunny days and tourists here get less sunshine. Therefore, Chengdu and Chongqing are chosen as the sampling places with high incidence of SAD winter symptoms. SAD tourists from Chengdu and Chongqing naturally have higher requirements for the sunshine environment. Sunshine factors will be mainly considered in their choice of winter rehabilitation tourism destination. In this study, sampling points were set up, respectively, in Chengdu of Sichuan and Chongqing and their surrounding areas. The scale was distributed by 12 trained volunteers. The data were collected twice by double-blind method (the first in December 2020 and the second in January 2021). At the same time, sampling points were set up in Haikou, Wenchang, Sanya, and the relevant surrounding areas in Hainan Province. Data were collected twice and 815 questionnaires were returned.

### Data collection, the inclusion and analysis of SAD standards

As the epidemic control increased the difficulty of sample collection, some tourists could only fill in the form on line by questionnaire star. After completing the pre-test content of the scale, the same tourist would fill in the post-test content of the scale again at an interval of 1 month. The results are shown in Table 2. Firstly, 6 non-SAD tourists were excluded from the total the 815 samples. Then, 114 samples with basic diseases and from non-sampling places were excluded from the remaining 809 samples. Finally, 695 samples meeting the requirements were determined. The composition of 695 SAD tourists included in the inclusion criteria was analyzed.

**Table 2:** Statistics of tourists with SAD.

Including standard (points)	Sample inclusion (excluding non-sample areas/underlying diseases) (*n* = 695)					
	RTM samples *n* = 376	SAD proportion	Non RTM samples *n* = 319	SAD proportion	SAD total	SAD proportion
0:0 < *X* ≥ 10:not SAD	0	0%	0	0%	0	0%
1:10 < *X* ≥ 15:mild SAD	110	29.25%	47	14.73%	157	22.59%
2:15 < *X* ≥ 20:moderate SAD	159	42.28%	194	60.82%	353	50.79%
3:20 < *X* ≥ 25:severe SAD	82	21.81%	76	23.82%	158	22.74%
4:25 < *X* ≥ 30:extreme SAD	25	6.66%	2	0.63%	27	3.88%

### Descriptive analysis

Women account for 67.0% and men 33.0% in these SAD samples. The population aged 20 and below, 21–30, 31–40, 41–50, 51–60, and over 60, respectively, accounts for 11.8, 42.7, 18.4, 14.8, 8.2, and 4.1% in the age distribution. The population rate of graduate, undergraduate, junior college and below are, respectively, 22.5, 68.0, and 9.5% in education distribution. Students accounted for 47.5%, professional and technical personnel 29.1%, private enterprise personnel 12.3%, government servants 3.4%, and other occupations 7.7% in terms of occupation. The number and prevalence of SAD tourists who travel in winter to Hainan for rehabilitation are similar to non-rehabilitation tourists in Hainan.

### The reliability and validity test of the scale

In this paper, the statistical KMO value and Bartlett’s spherical test were used to test the reliability and validity of the 695 scales of winter symptoms of SAD finally included in the study. The results are shown in Table 3.

**Table 3:** Results of reliability and validity analysis of the SAD Winter Symptom tourists Scale.

Variables	Cronbacha’s *α*	KMO	*χ* ^2^	*df*	*p*
SAD winter symptoms tourist QOL pre-test	0.754	0.826	2404.44	190	0.000
SAD winter symptoms tourist QOL post-test	0.770	0.818	2557.62	190	0.000
SAD winter symptoms observation group QOL pre-test	0.760	0.790	75.953	190	0.000
SAD winter symptoms control group QOL pre-test	0.772	0.864	38.119	190	0.000
SAD winter symptoms observation group QOL post-test	0.789	0.812	94.991	190	0.000
SAD winter symptoms control group QOL post-test	0.714	0.745	39.197	190	0.000

First, the reliability of the scale was analyzed. The results showed that the Cronbacha’s *α* coefficient value of the QOL pre-test (physical health, mental health, and social health) was 0.754, and the Cronbacha’s *α* coefficient value of the QOL post-test (physical health, mental health, and social health) was 0.770. Among them, the Cronbacha’s *α* coefficient value of the QOL pre-test of SAD winter symptom observation group was 0.760, the Cronbacha’s *α* coefficient value of the QOL post-test was 0.789, and the Cronbacha’s *α* coefficient value of the QOL pre-test of SAD winter symptom control group was 0.772. The Cronbacha’s *α* coefficient of the QOL post-test was 0.714. Therefore, it can be considered that the internal consistency of the set of test scales is good.

Secondly, the validity of the scale was analyzed. According to Kline (2011), the KMO value measurement standard of the scale is usually above 0.9, which is very suitable. 0.8 indicates fit; 0.7 means average; 0.6 is not suitable; Less than 0.5 is not suitable. The results showed that the KMO value of the QOL pre-test (physical health, mental health, and social health) scale for tourists with winter symptoms of SAD was 0.826, and the KMO value of the QOL post-test (physical health, mental health, and social health) scale for tourists with winter symptoms of SAD was 0.818. Among them, the KMO value of the QOL pre-test scale in the SAD winter symptom observation group was 0.790, and the KMO value of the QOL post-test scale was 0.812. The KMO value of the QOL pre-test scale in the SAD winter symptom control group was 0.864, and the KMO value of the QOL post-test scale was 0.745. The KMO values of the full scale were all above 0.7, and *p* < 0.001, the results showed that the scale had good internal validity.

### Hypothesis test

#### The normality test

Statistical spss20.0 statistical software is adopted to test the normality of 695 samples included. The pre-test skewness value of the sample data is –0.317 (skewness value >3 is regarded as extreme skewness), kurtosis value is 2.704 (kurtosis > 10 is regarded as a problem), and the value of Kolmogorov Smirnov statistic of the sample data is 0.62 (*p* < 0.5). Therefore, it is judged that the pre-test value of the sample is non-normal distribution. The skewness of the sample data are –0.241 and kurtosis 0.479, and the Kolmogorov Smirnov statistic of the sample data is 0.75 (*p* < 0.5). Therefore, it is judged that the post-test value of the sample is non-normal distribution.

#### Difference test

Six hundred ninty-five samples are grouped according to whether they travel to Hainan for rehabilitative mobility. One group is rehabilitative mobility SAD tourists in Hainan named as the observation group with a total of 376 cases; The other group is the non-rehabilitative mobility SAD tourists in Hainan named as the control group, with a total of 319 cases. Non-parametric Wilcoxon signed-ranks test was conducted on the pre-test and post-test values of physical health, mental health and social health indicators of tourists in the observation group. The pre-test and post-test differences within the group were analyzed. Within the 95% confidence interval, *p* < 0.05 will prove that the difference is statistically significant. The results are shown in Tables 4, 5.

**Table 4:** Difference test statistics between pre-test and post-test in observation group (*n* = 695).

Observation group				
Indicators	Pre-test and post-test were paired with Wilcoxon test			
	Pretest mean	Post-test mean	*Z*-value	*P* value
*Physical health*	30.7713	29.3163	−3.398	0.001
Drowsiness	3.25	2.96	−3.458	0.001
Weight	3.13	2.96	−3.112	0.002
Appetite	2.95	2.97	−0.214	0.830
Energy	3.10	2.54	−5.865	0.000
Flow breath	2.71	2.61	−1.100	0.271
Defecation times weekly	3.28	3.15	−1.680	0.093
Urination times weekly	3.21	3.04	−2.602	0.009
Concentration	2.97	2.65	−3.705	0.000
Memory	3.13	3.01	−1.445	0.148
Responsiveness	3.04	2.73	−4.763	0.000
*Mental health*	15.3590	14.0559	−5.976	0.000
Emotion	3.07	2.75	−3.638	0.000
Anxiety	3.09	2.74	−4.338	0.000
Happiness	3.09	2.67	−5.513	0.000
Interest in work	3.10	2.76	−4.453	0.000
Attention health	3.01	2.64	−5.365	0.000
*Social health*	15.0612	14.4453	−2.933	0.003
Social interaction	2.89	2.99	−1.538	0.124
Irritability	3.16	2.90	−3.597	0.000
Sensitivity	2.95	2.91	−0.689	0.491
Attitude of talking	3.02	2.99	−0.047	0.963
Cooperative commitment	3.03	2.67	−4.797	0.000
QOL	61.1915	57.840	−4.361	0.000

**Table 5:** Difference test statistics between observation group and control group a (*n* = 695).

Observation group and control group				
Indicators	Pre-test and post-test were paired with Wilcoxon test			
	the mean value of control group	The mean value of observation group	*Z*-value	*p* value
*Physical health*	30.9626	29.6216	−5.071	0.000
Drowsiness	3.41	3.19	−6.654	0.000
Weight	3.22	3.07	−3.889	0.000
Appetite	2.96	2.87	−2.630	0.009
Energy	3.21	3.35	−7.306	0.000
Flow breath	2.55	2.39	−4.728	0.000
Defecation times weekly	3.24	3.14	−0.200	0.842
Urination times weekly	3.05	3.01	−0.538	0.591
Concentration	3.05	3.11	−5.473	0.000
Memory	3.18	3.05	−1.306	0.192
Responsiveness	3.08	2.86	−5.263	0.000
*Mental health*	14.1309	14.1309	−4.134	0.000
Emotion	3.15	2.98	−4.363	0.000
Anxiety	3.14	2.98	−3.448	0.001
Happiness	3.08	2.72	−4.197	0.000
Interest in work	3.09	2.77	−1.281	0.000
Attention health	2.84	2.59	−0.027	0.978
*Social health*	14.9338	14.3871	−0.339	0.771
Social interaction	2.91	2.93	−1.343	0.179
Irritability	3.14	2.92	−1.549	0.121
Sensitivity	2.96	2.86	−0.811	0.417
Attitude of talking	2.95	2.97	−0.444	0.657
Cooperative commitment	2.98	2.71	−3.546	0.000
*QOL*	30.93	50.84	−6.155	0.000

## Results discussion

### Analysis of difference test results

#### The statistical results of the pre-test and post-test within the observation group

Tables 4, 5 shows *p* < 0.05 for the score of QOL (physical health, mental health, and social health) of the observation group tourists in the pre-test and post-test, indicating that within the 95% confidence interval, QOL of tourists in this group after rehabilitative mobility to Hainan has statistical differences in the dimensions of physical health, mental health, and social health. In addition, the post-test score of the observation group is lower than the pre-test score. The lower the score of QOL, the better the health of the tourists. Therefore, RTM to Hainan in winter can be regarded as having a therapeutic effect on QOL of SAD tourists. The results fully verify H1–H3.

However, Tables 4, 5 show *p* > 0.05 for the index scores of “appetite, smooth breathing, memory, and defecation times per week” in physical health and “social communication, sensitivity, and conversation attitude” in social health, indicating that these indexes have no statistical difference within the 95% confidence interval. There are several reasons why the index difference is insignificant. On the one hand, being stable in human physical health assessment, these indicators of “memory and defecation times per week” are difficult to change in a short period with the change of environment. On the other hand, the sample collection was conducted during the epidemic period and tourists were used to wearing masks to participate in activities on any occasion. Due to the limitation of social distancing and epidemic control, there is no significant difference in the above indicators of tourists in the observation group.

#### Statistical results of differences between the observation and control group

Tables 4, 5 show *p* < 0.05 for the score of tourist QOL (physical health, mental health, and social health) in the control study between the observation group and the control group, indicating that within the 95% confidence interval, there are statistical differences between the two groups in terms of physical health, mental health and social health. In addition, the QOL score of observation group tourists is lower than that of the control group. The lower QOL score, the better the health status of tourists. Therefore, RTM to Hainan in winter can be considered as having a therapeutic effect on QOL of SAD tourists, and the results fully verify H1–H3.

However, Tables 4, 5 show *p* > 0.05 for the physical health indicators “appetite, weekly defecation, weekly urination, and memory,” the mental health indicators “attention to health” and the social health indicators “social communication, irritability, sensitivity, and conversation attitude” of the two groups, indicating that there is no statistical difference between the two groups within the 95% confidence interval. There are several reasons why the index difference is insignificant. Firstly, in terms of “appetite,” as the sampling places are Chengdu and Chongqing, where people have eating habits and heavy taste preferences for strong Sichuan Cuisine, tourists do not adapt to the seafood and light diet in Hainan; Secondly, the indicators of “memory, weekly urination, and weekly defecation” are stable physical indicators. They are usually less affected by external factors and do not change in a short time, so there is no significant difference in the results. Finally, the sample collection was conducted during the epidemic period. The two groups of tourists were limited by the social distancing and the epidemic control to some degrees. When talking with people, they would keep the basic epidemic prevention distance. Tourists would become more sensitive and began to care about their words and deeds. Therefore, there is no statistical difference in these indicators.

**Figure 2:**
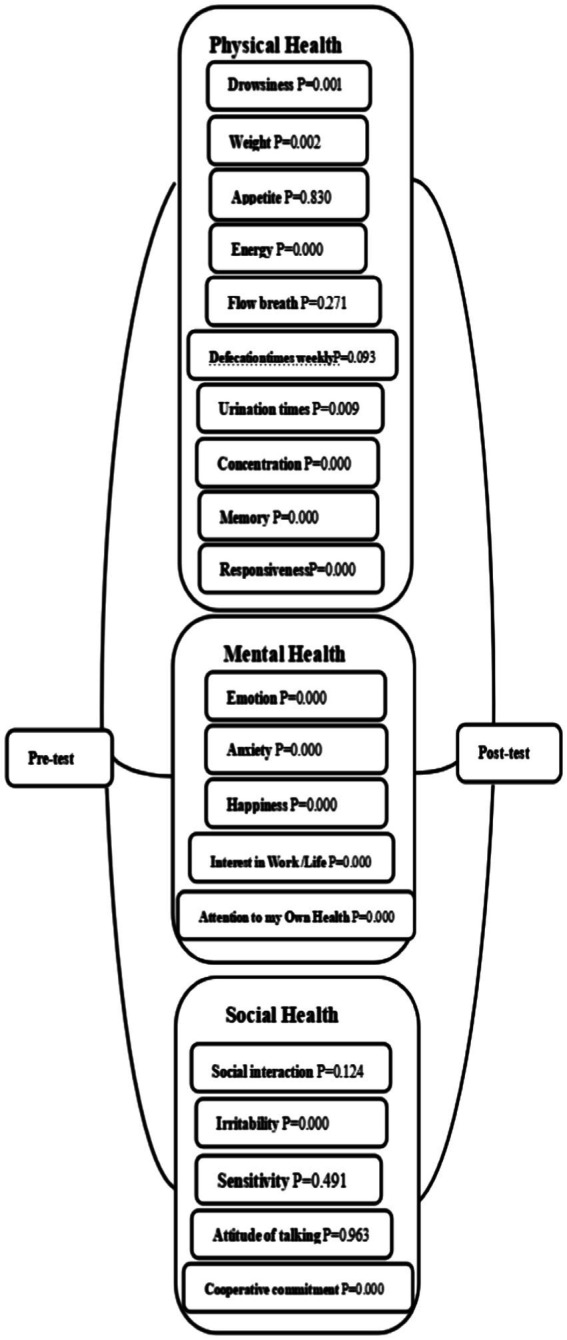
Statistical analysis of pre-test and post-test of observation group.

**Figure 3:**
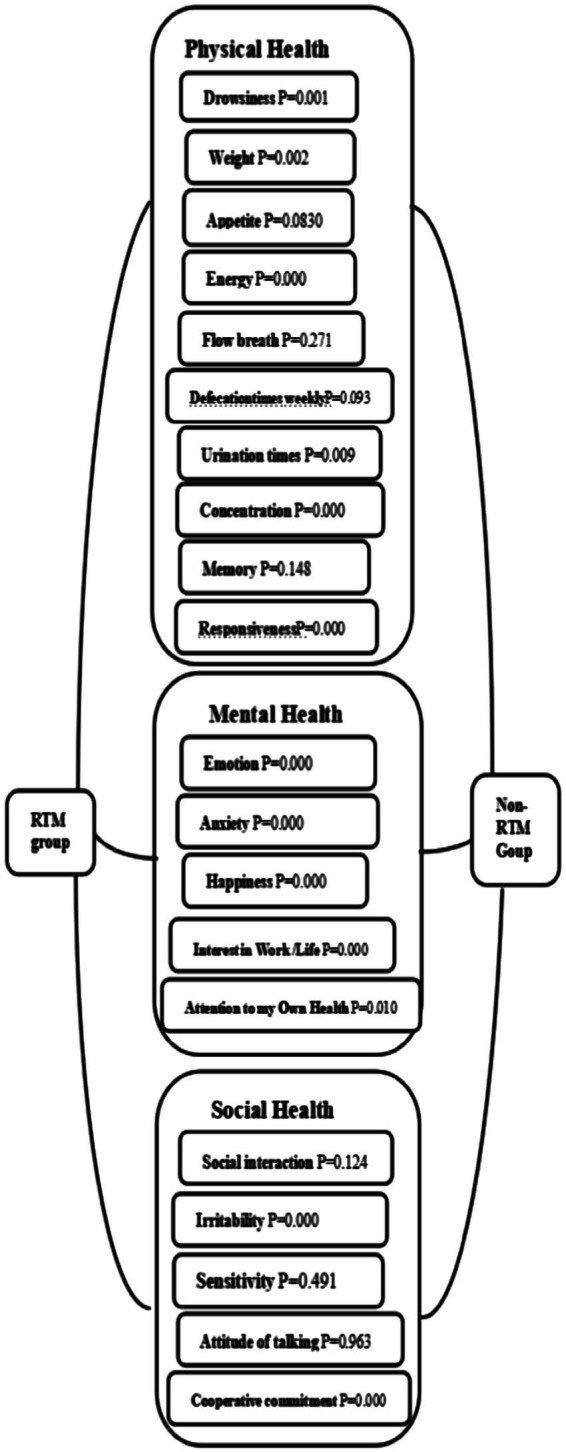
Statistical analysis of observation and control group.

The results showed that under the sunshine level provided by Hainan in winter, there were significant differences in the quality of life of the observation group and the control group in the three dimensions of physical health, mental health, and social health. This is shown in Figures 2, 3.

**Figure 4:**
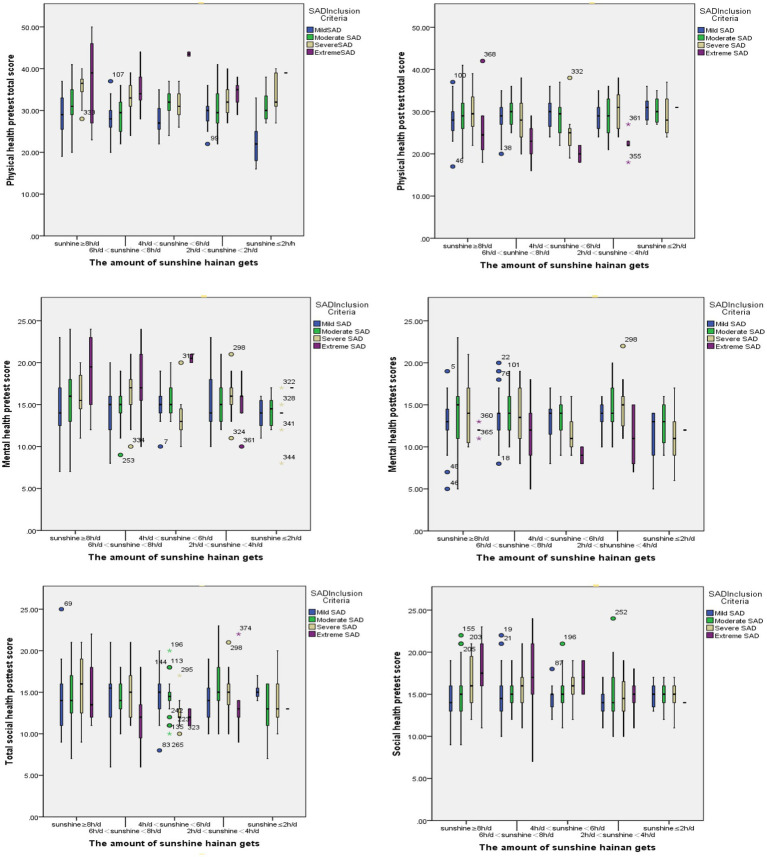
The effect observation of different degrees of SAD tourists’ QOL in different sunshine levels Hainan.

Taking sunshine level as the grouping variable, visual expression was used to show the rehabilitation effect of quality of life of tourists in the Hainan rehabilitation mobility observation group under different sunshine levels in Hainan from three dimensions of physical health, mental health, and social health. As shown in Figure 4, the higher the vertical axis score, the more serious the SAD symptoms and the poorer the therapeutic effect; On the contrary, the lower the vertical axis score, the lighter the SAD symptoms and the better the therapeutic effect. It can be seen from the Figure 4 that the pre-test scores of QOL of the observation group are lower than the post-test scores, indicating that RTM behavior can help SAD tourists to achieve varying degrees of rehabilitative improvement. Especially for the extremely severe SAD tourists, the physical health rehabilitative practices have the most significant improvement effect.

### Multivariate analysis of variance

Multivariate analysis of variance is an analysis of variance to determine whether an independent variable is affected by one or more factors or variables. For living in 376 patients with of SAD winter symptoms RTM observation group of tourists in Hainan to carry out multi-factor analysis of variance, testing different age, gender, Hainan sunshine level tourist group mean differences between the results of the variables, the observation group of tourists in Hainan living former health (physical, psychological, and social), and a month after the health (physical, psychological, and social) respectively as the result variable, Using multivariate analysis of variance of 2 × 2 × 2 × 3 on the improvement of the SAD winter symptoms tourists health situation, analysis of SAD RTM observation group tourists in Hainan health improvement is influenced by age, gender, and the influence of number of sunshine in Hainan, and analysis between these three factors, as well as the interaction between variables and covariate factors produce (see Table 6).

**Table 6:** Variance analysis of SAD winter symptoms RMT observation group (*n* = 376).

Variable source	Pretest			Post-test		
	Physical health	Mental health	Social health	Physical health	Mental health	Social health
Gender	8.101^**^	4.416^*^	0.082	0.026	0.771	0.844
Age	0.875	0.878	0.782	1.331	1.289	0.835
SAD include	4.121^**^	0.933	1.970	4.426^**^	2.386	4.057^**^
Sunshine level	2.787^*^	1.718	0.875	0.538	1.056	0.460
Gender*age	1.198	1.838	0.313	1.079	0.954	0.952
Gender*SAD include	3.077^*^	1.230	0.284	0.448	1.723	2.611
Gender*Sunshine level	0.851	0.448	1.372	0.518	0.703	1.723
Age*SAD include	0.531	1.114	1.018	1.168	1.633	0.779
Age*Sunshine level	0.559	0.767	0.480	0.921	1.548^*^	0.793
SAD include*Sunshine level	0.554	1.404	0.772	0.853	2.439^**^	0.805
Gender*age*SAD include	0.320	1.463	0.352	0.271	0.927	0.503
Gender*age*Sunshine level	0.772	0.821	0.420	0.489	0.446	2.351^*^
Age*SAD include*Sunshine level	0.766	0.677	0.445	0.380	1.462	0.666
Age*gender*SAD include*Sunshine level	1.019	1.855	0.118	0.477	1.182	1.015

*p < 0.05, **p < 0.01, ***p < 0.001.

As can be seen from table, the main effect of “gender” in the pretest physical health dimension of the Hainan RTM observation group of SAD winter symptoms in Chengdu and Chongqing was significant, *F* = 8.101, *p* < 0.01, the main effect of “SAD inclusion grade” was significant, *F* = 4.121, *p* < 0.01, the main effect of “sunshine number gained in Hainan” was significant, *F* = 2.787, *p* < 0.01, “gender *SAD inclusion rank interaction effect” was significant, *F* = 3.077, *p* < 0.05, indicating that the interaction effects of “gender, SAD inclusion level, and gender *SAD inclusion level” of migratory bird tourists with winter symptoms of SAD will have a significant effect on the improvement of their physical health. In the pretest mental health dimension, the main effect of “gender” was significant (*F* = 4.416, *p* < 0.05). The results indicate that the gender of the migratory bird tourists with winter symptoms of SAD has a significant effect on the improvement of their mental health. In the pretest social health dimension, the main effect and interaction were not significant, indicating that the interaction effect of “gender,” “SAD inclusion level,” and “gender *SAD inclusion level” did not have a significant effect on the improvement of social health of SAD winter symptom migratory bird visitors.

The physical health dimension, the main effect of “SAD inclusion level” was significant in the post-test (*F* = 4.426, *p* < 0.01; The results indicate that the inclusion level of SAD in Hainan RMT tourists has a significant effect on the improvement of physical health). In the dimension of mental health, the interaction effect of “age * number of sunshine gained in Hainan” was significant (*F* = 1.548, *p* < 0.05; These results indicated that “age” and “the number of sunshine in Hainan” had a significant effect on the improvement of mental health of the Hainan RMT group with winter SAD symptoms in Chengdu-Chongqing). In the dimension of social health, the main effect of “inclusion level of SAD” was significant (*F* = 4.057, *p* < 0.01, “gender * age * sunshine number in Hainan” interaction effect was significant, *F* = 2.351, *p* < 0.01). These results indicated that the interaction effect of “SAD inclusion level,” “the number of sunshine in Hainan, the gender and age of tourists” would have a significant effect on the improvement of social health of Hainan RMT tourists with SAD winter symptoms in Chengdu and Chongqing.

## Research conclusion, theoretical contributions and enlightenment

### Research conclusion

Since the occurrence of SAD is closely related to the short sunshine time in winter, the lack of light is considered to be the biggest cause of SAD (Wehr et al., 2001). Therefore, Rosenthal NE proposes that light treatment is the most effective and direct means for the treatment of SAD (Virk et al., 2009). Light therapy is believed to be able to solve the biological rhythm and mood problems of SAD and light regulation can significantly regulate the secretion of melatonin (Lam et al., 2001) with a rapid short-term effect (Cheng and Zhang, 2010) and a certain effect on the improvement of dietary disorders (Lam and Tam, 2009). The use of “light therapy” has become an alternative to antidepressants and psychotherapy (Tuunainen et al., 2004). Researchers have verified the improvement effect on SAD tourists after 30–45 min of irradiation with 10,000 lux light (Danilenko, 2007) and found that light therapy as a non-invasive physical therapy has very minor side effects (Lam et al., 2006) Danilenko mentioned that light therapy may enable SAD tourists participate in more autonomous activities (Zhai et al., 2020), and it has become the main treatment for SAD depression due to its advantages of effectiveness, convenience, safety and light side effects (Blashko et al., 1999). However, clinical studies of SAD on the standard, type, duration and experimental contrast of light therapy are still needed to be explored continuously. This study combines the method of follow-up interview with observation experiment, further demonstrating that the anxiety improvement effect of Migratory Bird Tourists with SAD winter symptoms in Chengdu city and Chongqing city is closely related to the length of sunshine time in winter.

The aim of this study is to reflect on the paradigms, opportunities and challenges of mobility research in the post-epidemic period, integrate interdisciplinary and interdisciplinary research resources, quantify the health effect of RTM on the human body, and think about opening a new understanding of mobility relationship. Compared with previous studies, first, careful selection of research objects, high sample quality, and minimization of confounding factors effectively avoid research bias in this research. Second, according to the standard score of the included samples, the winter symptoms of SAD were divided into four grades according to the severity of symptoms: mild, moderate, severe and very severe. The specific effects of sun exposure on the improvement of QOL of SAD tourists were evaluated by classification and stratification in this paper. Third, previous studies only pointed out that light can improve the symptoms of SAD patients, but did not deeply study the specific duration of light, and also gave specific light prescription for the rehabilitation of SAD tourists. In this paper, the light duration of SAD tourists in the cold shelter and mobile place was discussed in depth, and the effects of different sunshine levels on the physical, mental and social health of SAD tourists were scientifically quantified. It provides a valuable reference for scientific quantitative research of scholars’ hierarchy.

Firstly, the study finds that, with the sunshine exposure time in Hainan ≥8 h and 4 h ≤ the sunshine exposure time in Hainan <6 h, there is better improving effect on the physical health dimension of the observation group and the improved indicators are “responsiveness” and “concentration.” It shows that in the rehabilitative tourism environment in Hainan in winter, different sunshine exposure levels have a positive therapeutic effect on various indicators of human physical health. Secondly, the environment of RTM in Hainan also has a therapeutic impact on the mental health of tourists in the SAD observation group. In the dimension of mental health, the index coefficients are all positive and very similar, indicating that the therapeutic impact of each index of mental health is relatively similar. Thirdly, the supportive environment of RTM in Hainan also has an obvious therapeutic effect on the social health of tourists in the SAD observation group. The five groups of different sunshine exposure levels have similar effect coefficients on the tourists’ social health. With 4 h ≤ sunshine exposure time < 6 h, there is the best improving effect on tourists’ social health dimension, followed by sunshine exposure ≥8 h and 6 h ≤ sunshine exposure time < 8 h.

To sum up, the study discovers that RTM to Hainan in winter can create more sunshine exposure environment for SAD tourists, and provide a healthy field environment to support the improvement of QOL of this kind of sub-health tourists. The research results have verified the therapeutic effect of RTM on the improvement of the health and QOL of sub-health tourists. Based on this study, this paper puts forward the concept of “tourism rehabilitation,” and defines it as the comprehensive effect of health benefits when tourists go for RTM, stay in the healthy supporting field environment of seasonal tourism, personally participate in tourism activities and obtain situational experiences such as pleasure, relaxation and mobility.

RTM effectively connects the system of “place” of rehabilitation tourism and “person” of sub-health tourists. It can benefit the human body with positive physical health performance. Especially for SAD tourists, sunshine has become the regulating variable and the main factor for their health improvement. Tourists are “being there” (emphasizing the presence of people and technology) in the field of “the earth,” which supports the climate environment and special resources for health recovery. Thus the matter energy of “the earth” and “human” can be fully interactive, effectively supplement the life energy of the human body, realize the complementarity of “Yin and Yang” described in Traditional Chinese Medicine, and achieve physical health in the pleasant experience of appropriate movement and stillness. Based on people’s cognition, emotion and practice of RTM, people can produce positive empathy consciousness and psychological hint in the health support field of rehabilitation “place.” Positive ideology and hint will promote the formation of human mindfulness consciousness and transform the inner positive emotion into a good state of mental health.

### Theoretical contributions

As an exploratory research, this paper has paid attention to the unique characteristics of sub-health tourists and verified the therapeutic effect of RTM on them. Furthermore, it has clarified the mechanism of RTM on tourists’ physical and mental rehabilitation.

Firstly, this study aimed at the pathogenesis of tourist seasonal affective syndrome, scientifically quantified the effect of tourism treatment on the improvement of quality of life of tourists to Hainan RTM in winter, and explained the mechanism of action of rehabilitation tourism destinations on the improvement of quality of life. The study of mobility is no longer limited to common real-life scenarios and research fields, such as social identity, mental health, well-being, and self-concept. This study promotes the two-way knowledge spillover of the integration of tourism discipline and interdisciplinary research, and expands a certain space for related research.

Second, this study further explores how this is beneficial to human body health of environment field through the flow of people pursue active health rehabilitation effectively connected to the “people” and “to” system, realize the “ground”, and “people” the energy of interaction between cure tourists of body and mind, resulting in a positive psychological hint, pursuing the true self, improve the quality of life. This study expands the existing interdisciplinary research paradigm of tourism and provides more accurate experimental inclusion criteria and measurement methods for scholars to further study health tourism.

Finally, this study discovers the main factors affecting the therapeutic effect on tourists in RTM - sunshine exposure level. As the occurrence of SAD is closely related to the short sunshine time in winter, the lack of sunshine exposure is considered to be the biggest cause of seasonal emotional disorder. Sunshine plays a key role in creating a healing space for such sub-health tourists to improve their QOL. Through travel mobility, SAD tourists can get more health support than that in their usual place of residence. The sunshine environment helps to stimulate the production of human serotonin and the secretion of melatonin, effectively regulates biological rhythm and improves mood, and provides a valuable reference for the promotion and application of sunshine therapy, an alternative treatment for SAD winter symptoms. The research conclusion is expected to provide a scientific basis for the selection of rehabilitation tourism destinations for SAD sub-health tourists.

Limited by the seasonal sampling and interdisciplinary research, the universality and quantity of samples need to be expanded and increased. In the follow-up, more researchers are needed to expand the theoretical basis of tourism rehabilitation by increasing the sample size and long-term continuous tracing and observation. In the future, with the integration of cognitive neuroscience, bio-medicine, health technology, intelligent devices and other fields, it is expected to further explore the challenges and difficulties caused by “mobility.” The researchers can actively pay attention to what internal and external factors regulate the impact of tourism mobility on personal health, and think about what environmental characteristics help to improve human health performance and how RTM plays a positive role. With the help of the combining perspectives of medical sociology, health geography, physical geography and emotional geography, this paper interprets the function of tourism mobility on the improvement of human QOL, to provide a theoretical basis for health intervention in the process of travel mobility.

The research team plans to use the medical instrument EEG to monitor the dynamic effect of tourism rehabilitation on the existing samples by electroencephalogram imaging, so that the technical intervention can form a continuous one and remodel the trend of human health. A more rigorous and scientific theoretical understanding is expected to be achieved through follow-up research. The tourism therapeutic mechanism and research topics brought by RTM need to be clearly explained. People with a healthy physique will enjoy better experience of travel mobility.

### Practical enlightenment

The modern medicine-based disease treatment methods are easy to have side effects on the human body. The study of rehabilitation flow in appropriate seasons has a certain guiding role in the maintenance of human health and the prevention and treatment of diseases. Academic circles in recent years is focused on the health tourism development of relevant industries, focus on tourist flow impact on the health of the human body, even with all kinds of scale tests the mental health impact of tourism to tourists, subsequently and produce such as nerve tourism research, through the brain all and eye movement instrument equipment, such as for tourism irritants in the cognition of human’s emotion. However, existing studies are still difficult to clearly explain the mechanism of the health effects of tourism mobility. Moreover, due to the limitations of observation conditions and monitoring methods, it is difficult to continuously track and verify the health effects of tourism mobility on the human body. At present, the academic circles of tourism activities can affect the body’s physical health is still not clear, whether can to tourism as an alternative to drugs or medical intervention treatment human health, still to be further professional research, therefore, in this paper, as an exploratory study, also need to sample into the test, and the effect of cure SAD tourist for subsequent tracking, Into the future, will be to sample test before and after the introduction of medical instrument and electrical measuring contrast, through the brain electrical map to compare difference, as more clearly explain tourism bring natural light therapy cure effect of tourists, reduce inventory deviation, in order to get more seriously by later research and cognitive science theory.

This study promotes the integration of interdisciplinary research of tourism and life sciences. From the macro perspective, starting from the public’s market demand for health tourism, this paper explores how to realize effective tourism supply, provide with reliable theoretical support for resource development and product design for market suppliers. The study gives full play to the practical application of the mobility therapeutic function of rehabilitative tourism with the help of information tracing technologies, such as artificial intelligence wearable devices and big data. These will cater to the tourists’ pursuit of a healthy life and well-being. At the micro level, the study on the tourism therapeutic function brought by the rehabilitative tourism mobility will provide a more scientific basis for making a healthy tourism prescription, so as to promote the high-quality development of tourism industry.

## Data availability statement

The original contributions presented in the study are included in the article/supplementary material, further inquiries can be directed to the corresponding author.

## Ethics statement

Ethical review and approval was not required for the study on human participants in accordance with the local legislation and institutional requirements. Written informed consent from the (patients/participants OR patients/participants legal guardian/next of kin) was not required to participate in this study in accordance with the national legislation and the institutional requirements.

## Author contributions

SS contributed to the manuscript writing, data analysis, table and figure drawing and expert interviews. ZY contributed to Research design and logic sorting. WS contributed to literature sorting, paper translation and standardization, and manuscript submission and revision. LD contributed to questionnaire collection and language polishing. TL contributed questionnaire design, investigation, data collection and processing. All authors have read and approved the final version of the manuscript, and agree with the order of presentation of the authors.

## Conflict of interest

The authors declare that the research was conducted in the absence of any commercial or financial relationships that could be construed as a potential conflict of interest.

## Publisher’s note

All claims expressed in this article are solely those of the authors and do not necessarily represent those of their affiliated organizations, or those of the publisher, the editors and the reviewers. Any product that may be evaluated in this article, or claim that may be made by its manufacturer, is not guaranteed or endorsed by the publisher.

## Abbreviation

SAD: Seasonal affective disorder
QOL: Quality of life
GMDH: Group method of data handling
RTM: Rehabilitative travel mobility
RMSE: Root mean square error
MAPE: Mean absolute percentage error
MAE: Mean absolute error.
